# “Struggles and Falling Between the Cracks”: Experiences of Women With Disabilities on Using Intimate Partner Violence Services

**DOI:** 10.1177/10778012251379425

**Published:** 2025-09-25

**Authors:** Fredinah Namatovu, Veronica Lövgren, Kim Wickman

**Affiliations:** 1 Epidemiology and Global Health (EpiGH), Centre for Demographic and Ageing Research (CEDAR), 59588 Umeå University, Umeå, Sweden; 2 Department of Social Work, 8075 Umeå University, Umeå, Sweden; 3 Department of Education, 8075 Umeå University, Umeå, Sweden

**Keywords:** intimate partner violence, close relationships, disability service providers

## Abstract

Women with disabilities are at increased risk of intimate partner violence (IPV), yet their experiences of using IPV services are rarely studied. This qualitative study explored the experiences of 11 women with disabilities. Three themes were identified: “violence and disability—the continuums of stigma” describing the forms of stigma that women experienced that impacted the use of IPV services; “the dual nature of social relationships” summarizing aspects of interpersonal relations that influenced the use of services; and “to fall between the cracks” detailing institutional gaps that led to hesitation in using IPV services. These findings point at a need for increased effort to reduce stigma, capacity strengthening on disability-related issues and targeted resources to improve access to IPV services among women with disabilities.

## Background

Intimate partner violence (IPV) is considered the most common form of gender-based violence occurring at all societal levels and in most cultures around the world (Garcia-Moreno et al., 2006). IPV can manifest in several forms, including physical, sexual, psychological, and financial violence and abuse (Ellsberg et al., 2008; World Health Organization, 2013). Even though men experience IPV (Lövestad & Krantz, 2012), women are more likely to be victims of IPV due to the power structures that allow and reproduce marginalization (Garcia-Moreno et al., 2006). Several studies have consistently indicated that women with disabilities are more likely to be exposed to high rates of violence and abuse than women without disabilities (Hughes et al., 2012; Jones et al., 2012; Lund, 2011; Meyer et al., 2023; Valentine et al., 2019). In Sweden, just like most parts of the world, women with disabilities experience all forms of violence, from multiple perpetrators at different stages of their lives (Anyango et al., 2023).

The increased vulnerability of women with disabilities could be understood as a combined impact of disability and other axes of social marginalization, such as gender and socioeconomic status (Robinson et al., 2021). Additional contributing factors include dependence on others for long-term support and care (Brownridge, 2006; Nosek et al., 2001; Shah et al., 2016) and living in constrained social environments (Mueller et al., 2019). As Crenshaw (1991) highlights, the convergence of the intersecting forms of marginalization substantially amplifies the risk of violence and abuse.

IPV is not only a social and legal problem but is also considered a major public health threat that contributes to an increased risk of physical and mental health problems (Ellsberg et al., 2008; Garcia-Moreno et al., 2006; Namatovu et al., 2018; Thomas et al., 2008). Moreover, the devastating consequences of IPV tend to linger long even after an abusive relationship has ended (Anyango et al., 2023). It is therefore critical that IPV systems respond and take action to protect those exposed to violence, and to identify the needs of victims with disabilities given that they may experience multiple vulnerabilities (Evans & Feder, 2016; Rose et al., 2000).

Existing research on violence among women with disabilities has identified several important factors that IPV systems should take into consideration when responding to victims of violence with disabilities. The conceptual framework on access to healthcare by Levesque et al. (2013) suggests that to improve accessibility, services must be approachable, acceptable, available, appropriate, and affordable. Robinson et al. (2021) uses this framework to understand the alignment of the accessibility needs of IPV victims with disabilities and the actual violence-related services in place. Other key factors identified for bolstering access to violence-related services are the professional competence to facilitate trust, choice, and agency (Levesque et al., 2013; Robinson et al., 2021). Additionally, coordination and collaboration within the IPV system help to reduce the complexity that women with disabilities encounter when receiving support from multiple institutions (Klint et al., 2023; Namatovu et al., 2024). A Norwegian study noted the importance of raising awareness about rights and understanding the social and individual factors that influence the experiences of violence. Adopting this approach would enhance the effectiveness of interventions for people with disabilities (Åker et al., 2024).

Despite the documented benefits and reflections on how to organize support, evidence still suggests that in general, women with disabilities find it difficult to seek IPV services. This challenge is more pronounced among women with disabilities who face additional barriers due to their disabilities. Such challenges include infrastructural barriers (Feder et al., 2006; Robinson et al., 2021), lack of confidence in providers, and services not adapted to their needs (Evans & Feder, 2016). Obstacles in accessing IPV services often result in an increased risk of one's experience not being acknowledged, lack of information, or being unable to establish contact with other women in similar situations (Ellsberg et al., 2008; Garcia-Moreno et al., 2006; Skoog Waller, 2022; Thomas et al., 2008).

As previous research demonstrates, there is still a lack of evidence in Sweden regarding the nature of IPV services available and how women with disabilities experience these services. Research on IPV services in Sweden is based on the experiences of women in the general population. Most of this research investigates topics such as screening for IPV in healthcare facilities (Lawoko et al., 2011), seeking IPV-related services from social services (Dufort et al., 2013), and women's perceptions on health care utilization (Lövestad et al., 2021). Recent data from Sweden suggest that professionals lack knowledge and competence related to both violence and disability (Klint et al., 2023; Namatovu et al., 2024; Stark et al., 2024). Even though collaboration is considered critical, the system of collaboration is weak and differs substantially depending on the unit of referral (Klint et al., 2023).

This current study aimed to analyze the challenges that women with disabilities encounter in accessing IPV services. This study uses the term “IPV services” as an umbrella term for the various societal support structures and services available for people exposed to IPV (Namatovu & Ineland, 2024). This study specifically focused on the women's experiences of accessing IPV services that are offered by professionals within the Swedish welfare context working in healthcare, social services, police, women's shelters, and the Centre Against Violence. The IPV services offered include health care services, risk assessment, risk management, safety planning, support in making informed decisions, and building coping skills, safe housing, financial resources, legal and judicial support (Namatovu & Ineland, 2024).

### Theoretical Framework

Historically, people with disabilities have been regarded as “the other” and have been separated from the “normative” society because of their “otherness” (Kudlick, 2005). The process of othering creates and maintains a social order that produces or exacerbates discrimination, prejudice, and oppression (Goffman, 1959). The same process of “othering” results in discrimination and systemic exclusion of people with disabilities (Bogart & Dunn, 2019), affecting their level of social participation and access to services, including IPV services.

Discrimination against people with disabilities is so recurring and pervasive that even those assuming a progressive posture (un)consciously support ableism (McRuer, 2008). Ableism is a social prejudice towards people with disabilities, it is a network of beliefs, processes, and practices constructed around a norm of the able body, projected as perfect, typical, a body without physical or cognitive disability, impairment, or chronic illness. People of able body/mind construct the world, language, culture, and belief systems to maintain their norm as superior (McRuer, 2008). These ableist views support discrimination which creates and sustains other oppressive systems (Campbell, 2008).

The structures created to uphold these ableist attitudes interfere with the full and equitable participation of women with disabilities, eventually excluding them from accessing available services. The oppression resulting from ableism can occur not only at an institutional level, through laws and policies but also social norms, that might color the personal narratives of women with disabilities (Mueller et al., 2019). Stigma creates access barriers, some scholars conclude that women who have previously encountered negative experiences in accessing healthcare systems are less likely to reuse health systems or seek appropriate healthcare (Bowes & Domokos, 1993).

In this study, applying the ableist theory to the findings serves to expand our understanding of the experiences and perceptions of women with disabilities concerning their use of available IPV services.

## Methodology

This qualitative study was part of the DISIPV project, which assessed accessibility to IPV services among women with disabilities (Namatovu & Ineland, 2024). It draws from in-depth interviews with 11 women with disabilities who had experienced IPV and subsequently sought professional help. The authors approached the data from a constructivist epistemological lens cognizant that scientific knowledge is constructed and shaped by the social and cultural contexts within which we operate (Guba & Lincoln, 1994). We embraced reflexivity, aware that the research process is affected by our own subjective experiences and understanding of the world (Morrow, 2005). An experiential orientation to data interpretation was used to highlight the experiences, perceptions, attitudes, and opinions of women with disabilities on the challenges of accessing IPV services. By doing so, we were able to emphasize the meaning and meaningfulness as ascribed by the women. We therefore embrace our own socially constructed subjective meanings and those of our study participants (Guba & Lincoln, 1994).

### Data Collection

Purposive sampling was used to identify the study participants in a recruitment process that lasted approximately 10 months during the autumn and winter of 2020–2021. The recruitment efforts targeted both national and regional spaces used by women with disabilities, such as social media platforms for disability organizations, membership magazines, and spaces for all women shielding themselves from IPV, such as shelters in different regions of Sweden. Although the project invited both men and women with disabilities to participate, only women contacted the research team, which made them the primary focus of the study. Thirteen women expressed interest in participating, but only eleven were interviewed. The two who were not interviewed had hearing impairments and could not find a sign language interpreter due to the COVID-19 outbreak.

The inclusion criteria required participants to self-identify as having a disability and to have had experiences of seeking support concerning IPV. Participants were invited to share both negative and positive experiences of IPV services.

Regarding the participants’ characteristics, their age ranged from 25 to 60 years, all identified as women, four participants had children and five had half-time jobs. Additionally, all participants self-identified as having a disability. The participants were not asked to specify the nature or severity of their disability, although only those able to independently hold a conversation were interviewed. During the interviews participants spoke of having multiple disabilities. The disabilities reported were typically characterized by mobility impairment, hearing impairment, attention deficit hyperactivity disorder (ADHD), personality disorder, stress-related disorders, eating disorders, depression, anxiety disorders, and post-traumatic stress disorder.

The Researcher who conducted the interviews was highly experienced in interviewing people with disabilities and has been working in this field for nearly two decades. The interviewer remained attentive to the participants’ disabilities throughout the recruitment and the interview process, ensuring an inclusive and accommodating approach. Participants were given the opportunity to set the pace of the conversation. At the beginning of each interview, the participant was reminded to request breaks or end the interview if needed. In some cases, interviews lasted longer when participants required pauses, needed to refocus, or engaged in tangential discussions before returning to the main topic. One participant requested to be interviewed while taking a walk, while another preferred a phone interview while walking in a public space. All requests were accommodated to create a comfortable and inclusive environment.

The length of the interviews ranged between 56 and 100 min. A semi-structured interview guide was meticulously crafted and subsequently employed for data collection. The interview guide covered a range of questions organized into four broad topics: (a) accessibility and contact with IPV services, (b) suitability of IPV services, (c) competence development and recommendations, and (d) experiences of IPV during COVID-19. For this analysis, we primarily focused on materials related to the first two categories. An interview guide is provided to show the specific questions included, Supplementary File 1. All interviews were audio recorded, transcribed verbatim, and later translated into English. The questions analyzed within the scope of this paper were centered on elucidating personal experiences with various IPV service providers.

### Data Analysis

Data analysis was conducted according to the six-phase process of reflective thematic analysis as outlined by Braun and Clarke (2006, 2019, 2021, 2022). The primary responsibility for data analysis was conducted by the first author. Consequently, the study findings reflect the first author's interpretation of the data. However, the second and third authors engaged in an ongoing dialogue with the first author through debriefing sessions providing critical feedback and interrogated the interpretations.

The first phase of familiarization with the data began with the first author repeatedly listening to the audio recordings and reading data transcripts several times. This process also involved consultation with the second author who conducted the interviews to gain more clarification when needed. In the second phase, data was imported into MAXQDA, a qualitative software that was used by the first author for initial data coding. In subsequent iterations, codes were modified, added or removed based on the author's interpretation of what was considered meaningful. Coding was primarily done using an inductive approach, with limited deductive reasoning to ensure that the identified codes were aligned with the study aim. In the third phase, the initial themes were assigned by reviewing all codes and grouping them into broader topics. Themes were primarily developed at a semantic level to report the surface meanings of the participants’ descriptions (Braun & Clarke, 2006, 2019, 2021). In Phase 4, the identified codes, themes and subthemes were exported into a Microsoft Excel spreadsheet for further analysis. All co-authors jointly interrogated the exported codes, themes and subthemes and several amendments were made. In Phase 5, the themes and subthemes were defined and renamed several times to ensure that they were concise and meaningful. In the sixth phase, the first author finalized the writing of the results, integrating all themes and subthemes into a coherent narrative. Even though the analysis phases are presented here chronologically, the actual process required moving back and forth, iteratively and reflectively as suggested by Braun and Clarke (2006). Table 1 provides an example of codes that informed the creation of theme two and its subthemes.

**Table 1.** Example of Codes and Subthemes Presented in Theme 2.

Codes	Subthemes	Theme 2
Why bother, it is almost impossible to get full custody	Weighing the cost of a violence free life	The dual nature of social relationships
Changing address affects the children's social life		
Loosing custody to one that abuses you and your children		
Your ex-husband framing you as a dangerous mother		
My friend knew who to contact, she had been a victim too	Social networks as removers of blinders and way makers	
I got encouraged by my mother and my friends		
My girlfriends they were my soldiers who protected me		
I was scared, my mother called the police, they came		
My friend in USA said I must leave		

## Results

**Figure 1.**
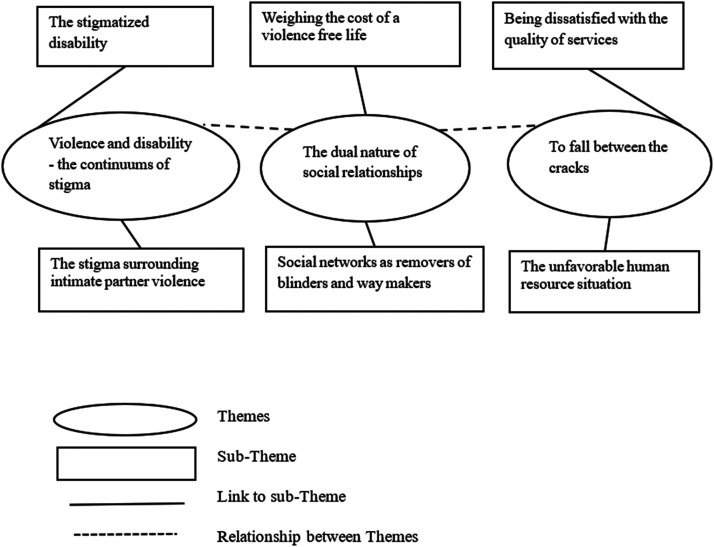
A Thematic Map Showing the Three Themes and Their Subthemes.

The analysis resulted in three overarching themes that encapsulate the challenges encountered by women with disabilities during utilization of IPV services. Figure 1 shows a thematic map of the themes and subthemes, followed by a detailed description of the results in the preceding text.

### Theme 1: Violence and Disability—the Continuums of Stigma

Several participants described IPV and disability as highly stigmatized. The two subthemes below illustrate how stigma impacted the women's utilization of IPV services.

#### Subtheme 1: The stigma surrounding intimate partner violence

Several interviewees also described living in a social context where IPV was still perceived as a taboo, something kept to oneself and not discussed with others even within a close family set up. This cultural background led to the internalization of stigma which affected women's willingness to talk about their IPV experiences. The anticipation of encountering stigma at the various institutions created hesitancy to seek professional help.

One woman observed: “Yeah, I did not say it until later, I hardly dared to say it” (Katarina).

The exposure to abuse coupled with societal silence created what is characterized as internal and external stigma (Goffman, 1963). Internal stigma, or self-stigma, occurs when women adopt and apply to themselves negative societal beliefs and attitudes directed towards them. In this study, women described a profound sense of shame, embarrassment, and self-blame for the abuse they endured. External stigma is the negative attitudes, beliefs and practices the women encountered from others directed towards them. It was rooted in societal perceptions and communicated by the women's social networks and the service providers. For example, Erica described how her mother's need to maintain a good social image prevented her from having conversations about her IPV experience. “My mother is of the generation that is a bit hush-hush, things should look good on the outside. But she is aware of what is happening” (Erica). Another woman felt that the stigma surrounding violence made it difficult for victims to report. “Abuse is quite a taboo here in Sweden and you never talk about it … those who dare to talk about it do not even dare to report it” (Tilda). This underscores the consequences of oppression where a woman subjected to IPV tend to feel less valuable than everyone else often resulting in a self-understanding of being unworthy. This affected women's willingness to talk about their IPV experiences and created hesitancy to seek professional help.

This subtheme summarizes how the stigma surrounding IPV shaped the women's understanding of IPV and their decision to seek professional help. At the time when IPV occurred, all the study participants were in heterosexual relationships where abuse was perpetrated by a male intimate partner. The normalization of men's harmful behavior hindered women from seeking formal support as exemplified in the quotations below.

He would watch everything I did, call me names, guilt-trip me about money, but no one saw that as abuse. It was always brushed aside … they told me that this is just the way men are in general. That I must adjust. I started to think that perhaps I was being too sensitive. (Theresa)

The gendered normalization of IPV as illustrated by Theresa was promoted by sexist prejudices that excused harmful male behavior as inevitable. Statements like, “*this is just the way men are”* illustrated power imbalance, where women were expected to tolerate the abuse at the expense of their safety and wellbeing. Normalizing abuse further re-enforced self-doubt where women questioned their own emotional response as—“*being too sensitive*.” Framing abusive behaviors as typical or unchangeable not only deterred women from acting by seeking for help, but it also implied that systems–such as the social and legal could not intervene. Additionally, the lack of social and legal consequences implied that perpetrators were not held accountable, therefore had no incentive stop the harmful behaviors.

#### Subtheme 2: The stigmatized disability

The stigma surrounding disability was another form of external stigma that women experienced that was directly related to their disability. This stigma was often encountered during the women's interaction with various institutions that provide IPV services. Some women with visible disabilities perceived that service providers focused more on their differences based on their disabilities than on the IPV they reported. When their disability was the most prominent aspect, it overshadowed their IPV experience, the very reason why they sought support. Because of their disability, service providers treated them with negative stereotypes, prejudiced attitudes, and discriminatory behavior, illustrated in this participant's explanation:

You just become synonymous with your diagnosis [disability] or your history. It very much becomes a pity. I have a hard time talking to people, e.g., counselors … doctors, those who have not worked with people who have gone through things…. I met a psychologist and a counselor who started crying. I sat there thinking this is my life, my everyday life, this is what I live with every day…. I was very pissed when I got there…. I was angry at everything and everyone. (Erica)

Erica perceived that her disability led to the loss of her identity because disability was a state considered undesirable by professionals which evoked pity. In anticipation of a stigmatizing encounter, some women adopted stigma management strategies to guard against being stigmatized. For example, some resorted to hiding their disabilities while others refrained from seeking IPV services.

In brief, the women who participated in this study experienced multiple layers of stigma attached to IPV, that did not always occur in isolation but was often compounded by the stigma associated with disability. Together, these overlapping forms of stigma shaped their experiences of IPV services. In the next theme, we reflect on how women viewed the role of social relationships in navigating IPV services.

### Theme 2: The Dual Nature of Social Relationships

This theme compiles the participants’ description of a paradoxical situation of the role of social networks in seeking IPV support which is presented in two subthemes.

#### Subtheme 1: Weighing the cost of a violence free life

Women expressed hesitance in seeking formal support because of its social and legal ramifications. Social relationships such as husband/partner, children, parents, and friends had both a negative and positive impact on the women's attempts to use IPV services. Married victims of IPV who considered seeking formal support found themselves caught at the crossroads. On one path, reporting IPV offered a pathway to leaving the abusive relationship, promising safety. On the other path, reporting IPV could lead to divorce, a state that was considered less desirable as noted by Jenny: “I did not want to slaughter a marriage” (Jenny). Jenny used the word “slaughter,” a rather strong word that evokes violent destruction, hence her hesitation to act due to the fear of bearing the social blame for causing irreparable harm to the marital relationship.

Additionally, many mothers who were victims of IPV found themselves weighing the price of safety from abuse against the impeding consequences. This difficult decision often involved balancing the immediate need for safety with the short-term and long-term consequences divorce had on children, making the choice to report abuse even more complex. Many carefully weighed the legal implications of divorce, dreading the potential of custody battles and the disruptions divorce could cause in the children's day-to-day living conditions. Jenny continued to narrate:

I wanted the children to be close to the school so that they would keep their friends, I don’t want to move away, and I know how difficult it is to get sole custody. It is almost impossible to get sole custody today, despite intimate-partner violence. (Jenny)

#### Subtheme 2: Social networks as removers of blinders and way makers

Another critical role of social relationships was helping women to identify and name various forms of violence. In retrospect, some participants described uncertainty regarding what counted as psychological violence at the time when this violence occurred. All the interviewed women recalled being knowledgeable of what constituted physical and sexual violence, a few recognized economic violence, however, most women had not encountered the term “psychological violence.” Despite the participants experiencing situations in which their partners exhibited controlling behavior, screaming, intimidation, they did not initially characterize these actions as abusive or warranting support. It was through conversations with others that they came to recognize that they had experienced psychological violence/abuse from their partners. According to Jenny: “They told me that I had been subjected to psychological, physical, and economic violence. At first, I did not know what psychological abuse was” (Jenny). Realizing for the first time that the treatment she had perceived as “normal” was not, gave her a new understanding and recognition that psychological violence is a form of IPV requiring support.

For some women, disclosure to their social networks helped them recognize the severity of the abuse, understand the extent of the danger they were in, and determine the course of action required. In many narratives, social networks played a pivotal role in encouraging women to seek IPV services. However, this was mainly dependent on the response a woman received after disclosing an experience of IPV to a close contact. For some women, this disclosure helped them to realize how serious the abuse was, or to understand how much danger they were in, and a need to seek formal help. “I talked a lot with both my mother and my best girlfriend who then lived in the USA. She said on several occasions that she was genuinely worried about me” (Cecilia). Cecilia's dialogue with her best friend helped highlight the magnitude of violence which led to seeking formal support.

Another important aspect of seeking IPV support is services being accessible. The participants narrated that to be able to access the comprehensive IPV services, this required contacting multiple institutions simultaneously. This process was viewed as complex and overwhelming. Women described that because of their disabilities it was often difficult to navigate a multifaced system on their own. They often appreciated support from a family member or friend: “I remembered that my mother and the legal representative worked a lot … mom had to call and report him (the abuser) to the police” (Katarina). In this material, it was particularly mothers who played a pivotal role in encouraging their daughters to seek IPV services. Mothers also helped to initiate contact and to accompany women to IPV service providers, a role that was highly appreciated by though who found it this challenging due to disability.

In summary, supportive family and friends played an important role in labeling violence and encouraging the women to seek IPV services, yet fear surrounding the consequences of ending a committed relationship made some women hesitant to involve professionals. The next theme explores factors at an organizational level that influenced seeking formal IPV services.

### Theme 3: To Fall Between the Cracks

This theme describes the complexity and gaps within the IPV system that led to women with disabilities “to fall between the cracks,” preventing them from accessing the care, services, and resources they needed. The first subtheme describes an unfavorable human resource situation, which was manifested through the long waiting duration and a high turnover. The second subtheme touches upon the barriers created by the imbalance in power relationships.

#### Subtheme 1: The unfavorable human resource situation

Some women were discouraged from seeking IPV services due to the IPV system's unfavorable human resource situation, which manifested as a long waiting duration and high turnover. The phrase “*to fall between the cracks*” was commonly used among our participants, which we interpreted as getting stuck in between the system without getting the actual help. Women not only questioned the effectiveness of the institutions that provide IPV services, but they were also critical of the government's ability to address the needs of people with disabilities. Here are some examples of how this was expressed among the women:

It becomes a very difficult thing…. You have a support system that is supposed to support you in such situations, but that does not happen…. So many fall between the cracks (Maria).

I have waited an extremely long time … it shouldn’t be the case that you have to wait 7–8 months to have psychological help … that's why I'm critical and skeptical of our government … you should not have to fall between the cracks because you do not get the help and support and care that you have asked for. (Theresa)

Because of insufficient personnel, some women felt that service providers were often in a hurry and acted impatiently which gave the women the impression that they wanted to get rid of them by referring them to someone else “We did not have time to talk at all. He just asked if he could send me on to a colleague” (Cecilia). The time allocated per client is based on the needs of an “able bodied” person, without accounting for situations where communication could be constrained by a disability. The urgency portrayed by the service providers conveyed a message that the women's needs were not important which led to further marginalization and exclusion. Sometimes marginalization occurred openly and directly while on other occasions it was indirect and subtle. Linda pointed out the problem of time constraints.

Each authority has a certain amount of time allocated to attend to clients, either for treatment offered free of charge or if clients must pay a fee. Once this time is over, the service providers do not accept to take on any more clients. In the case of courts, the prosecutors shut down. The district court closes. (Linda)

Operating under time-constraint forced service providers to end sessions prematurely. This created resentment from those who wanted longer sessions, which demotivated many from seeking these services.

The high turnover of service providers was another challenge described by the interviewed women. For the participants, this meant having to constantly re-introduce their problems to new staff members. Not only did they have to revisit the painful stories, but it also limited progress in getting the support, as this participant narrated. “You get sick between doctors…. Every time you come to psychiatry, you meet a new doctor, which means that you must tell your whole life story again. You never get anywhere” (Erica). Women who had encountered this slow progress perceived seeking professional help as tedious and ineffective, and thus demonstrated hesitance in future use of such services.

#### Subtheme 2: Being dissatisfied with the quality of services

The poor quality of IPV services was another factor that discouraged some women from seeking IPV services.

Confronting a system that was not always tailored to their specific disability-related needs created significant hurdles in navigating the IPV system. One example of encountering inadequate services was illustrated by a woman with a hearing impairment. In her attempt to seek support from multiple actors, she faced the challenge of communicating through telephone services, which were inaccessible to her.

I had a very hard time from the beginning when contacting the women's shelter because I have a hearing impairment, I find it difficult to talk on the phone…. I cried a lot because I could not hear. I even tried talking to the person responsible for disability issues at the Swedish Social Insurance Agency, but this was not helpful because this person spoke so quietly, I could not understand what was communicated. Then I decided to call another insurance company to see if they could make a phone call on my behalf. I explained that I needed this help because when I called on my own, they could not understand what I said. (Jenny)

One of the recent developments in Sweden is the use of mobile phones and digitalization in the delivery of services. As described in the text above, majority of the agencies mandate that the initial contact be made on the phone. However, this approach does not consider that this means of communication is not accessible to people with hearing impairments. In the example above, not only was the phone technique not adapted to enhance hearing, even the service provider was not mindful of the client's impairments as illustrated by talking very softly. Like Jenny, several women were excluded from support by the IPV system that was not adapted to their disability needs. Women with hearing impairments felt that systems should be adapted to enable communication with IPV service providers and government institutions regardless of their disabilities.

Another shortcoming was observed in professional conduct. Some women were discouraged by the professionals’ lack of empathy and understanding during their interactions. Professionals were described as cold and uncommunicative, only interested in following their professional routines. These experiences left women dissatisfied with the encounters with IPV service providers as Erica narrated:

I can meet a new psychologist and if the first thing they do is make a psychologist sound, then it doesn’t work. You want a conversation. I have not met a person who has been in contact with psychiatry who has not been disturbed by these fucking sounds. (Erica)

Erica, like many other women in this study, was critical of the communication style she perceived as overly clinical and detached. The human connection, characterized by relational engagement in professional relationships, was perceived as important in providing formal support. Women desired a genuine human interaction with a conversational tone, warmth, and empathy. When professionals adopted a clinical stance, such as making what Erica describes as “psychologist sounds,” it created discomfort, and a feeling that they were being analyzed, an approach they considered dehumanizing.

Tilda was appalled by a service provider who breached confidentiality by sharing her personal information without first obtaining her permission. “I was greatly disappointed because I felt that I was opening up to the last person now and throwing a stone in the hope that it would not sink as far as it could … yet she leaked my personal information” (Tilda). Some women had been in contact with service providers whom they perceived as not following the standard procedures, as the quote from Linda illustrate: “Despite the social services guidelines that are in place, no one follows them. Investigations are not done according to the right methodology or based on solid facts, but just opinions and their way of thinking” (Linda). It is important to underscore that a service provider who breaches confidentiality or who does not follow the proper protocol for reporting violence or abuse may not only violate the women's rights, lose their trust, and damage their reputation, but they may also put women at risk of further harm.

Additionally, some women described experiences a power imbalance that fostered institutional mistrust, deterring them from seeking support. Several participants perceived service providers as occupying a privileged position, acting as gatekeepers who determined which IPV services to offer and to whom. One respondent who had been in contact with the health care system for 11 years, shared her past experiences of violence and abuse and expected to receive support related to her past trauma that still affected her. Despite her continued pleas, the service providers continuously ignored her, prioritizing aspects they deemed more important.

I visited the healthcare hoping to talk about the sexual abuse I was subjected to in the past…. They asked what my problem was, and I told them that I suffered from panic disorder, I get severe stress. I would tell them that I had experienced all symptoms, from stress eczema to being burned out, various things. Then they would switch the topic and ask, “Has there been any trauma?”. Then I would talk about my trauma from the past. But then they switched to “But what's the problem today? That past trauma happed so long ago.” It took 11 years from when I was sexually assaulted until I received the PTSD treatment. (Cecilia)

The above excerpt illustrates a hierarchical power structure where a service provider occupies the superior position of deciding what is important to be addressed. Cecilia, like many other women who encountered similar treatment at numerous institutions, was left frustrated by encountering a deaf system that didn’t hear her as she struggled to explain her needs. They chose what was important for her. Such encounters made women hesitant to continue seeking IPV services. Examples of power imbalance were also illustrated in communication between the women and the service providers. Erica recalled being completely ignored by the professionals, because of her disability: “No one talked to me, they talked about me over my head…. I was told that the reason why we were there was because of me, but no one talked to me” (Erica). She and several other women had been in contact with service providers who discussed with each other and sometimes with the women's caretakers but did not include the women in the conversations. These left women yearning to be met with the basic human dignity they rightfully deserved as Erica remarked, “wishing to be treated like humans.”

Furthermore, lack of consequences for perpetrators discouraged many women with disabilities from reporting IPV. Women considered that reporting IPV would result in accountability for the perpetrators; however, this was not always the case. The absence of legal or social consequences for the perpetrators discouraged many victims and left them with many questions: what was the point of reporting violence/ abuse if there were no consequences to the abuser? This perception discouraged many women from reporting IPV.

Yeah, the only one I told at the beginning was my mom. She wanted me to report, and I could not…. I know how it works…. I know how many cases are closed. It felt unnecessary. I was just going to stick my head in the sand. (Cecilia)

Many participants interfaced with other inadequacies in the legal system where IPV victims were confronted with delays in the legal proceedings, lack of evidence, and insufficient enforcement of protective orders.

## Discussion

This qualitative study aimed at gaining insight into the challenges preventing women with disabilities from accessing IPV services. The identified challenges are presented in three key themes: (a) “violence and disability–the continuums of stigma,” (b) “the dual nature of social relationships,” and (c) “to fall between the cracks.”

Findings from this study indicate that stigma related to both IPV and disability discouraged women from seeking IPV services. This finding concurs with existing evidence showing that stigma towards IPV victims is a barrier to disclosure and help seeking (Murray et al., 2015). On the provider side, the stigma toward IPV victims negatively influences response and measures taken against aggressors (Murray et al., 2015). The stigma attached to disability occurring during interactions with service providers can be viewed as structural discrimination that primarily affects the marginalized groups (Heise, 1998). This type of stigma is instigated by service providers and is rooted in the biomedical perspective, where disability is perceived as a tragedy that needs to be eliminated (Coleman, 1990, 1997). This attitude within health care re-enforces discrimination directed towards people with disabilities (Kudlick, 2005; Thomas, 1999). Stigmatization of women with disabilities lowers their self-esteem and self-efficacy, resulting in hesitancy in seeking IPV services. When the stigma around IPV and disability intersect, they exacerbate discrimination, ostracism and professional inaction (Fleming & Franklin, 2021).

As illustrated in this study, in anticipation of stigma related to disability, some women disguised their disabilities when seeking IPV services. This phenomenon is discussed in the literature, where people with imperceptible disabilities resort to hiding them to evade discrimination and systemic preclusion (Cureton, 2018). Women with less discernable disabilities may employ a classic Goffman strategy of “passing” (Goffman, 1963), to hide their disabilities from the service providers. Even though passing as a form of self-protection is helpful in a moment, it fosters further disablement (Leary, 1999). It is important to note that when different sources of stigma collide a new set of barriers is created for those situated within the convergence of these marginalization forces. It is important to continue challenging the conditions that create and maintain stigma to promote the use of IPV services among women with disabilities.

Social relationship played a dual role, sometimes restricting but also enhancing the utilization of IPV services. Being married or in a relationship was depicted as a hindrance to seeking professional help. Even though married women recognized the danger of staying in a violent relationship, many were hesitant to seek professional help, fearing to be blamed for ending a marriage. Research indicates that survivors of IPV frequently encounter victim blaming attitudes. These attitudes unjustly place the blame for violence on the survivor, which significantly hinders the victim's willingness and ability to seek help (Meyer, 2016). Research further suggests that women with disabilities might choose to remain in abusive intimate relationships because these relationships tend to hold different meanings and to serve different purposes for those with disabilities (Pugh et al., 2021). Negative attitudes towards women with disabilities, such as be seen as undesirable, undermine self-esteem, and might leading women with disabilities to remain in abusive relationships due to fear of not finding another partner (Jóhannsdóttir et al., 2021). Custody concerns and the children's wellbeing following divorce were yet additional explanations for continuing in marriage despite the violence. Previous findings note that having children is a major reason why women stay in abusive relationships (Meyer, 2012).

Friends and family provide a range of support, including emotional, informational, and appraisal, which promotes seeking IPV services. This finding aligns with previous research showing that support from family and friends facilitates the decision-making process and empowers abused women to leave violent relationships (Skoog Waller, 2022). Additionally, support from social networks is essential in helping women with disabilities to navigate a complex and fragmented IPV system (Namatovu et al., 2024). In this study, some described the difficulty in recognizing psychological violence as a major hindrance to seeking the related support. Current evidence suggests that women in general find it difficult to recognize and name psychological abuse in the absence of physical abuse (Stark, 2007). The challenge of naming psychological violence is also common among professionals (Seff et al., 2008), as demonstrated by the lack of a consensus regarding behaviors that constitute psychological abuse (Follingstad & DeHart, 2000). The inconsitencies and ambiguity in characterizing psychological violence might be attributed to the depiction of violence in popular culture (Canfield, 2007), where violence is often portrayed as an explosive isolated event (Berns, 2001), making psychological violence which is less dramatic hard to recognize.

This study found that some women who attempted to access IPV services fell through the cracks due to insufficient human resources, power imbalances, and professional misconduct. The issue of insufficient human resources is well documented in Sweden, both the healthcare and social services are characterized by long waiting times and delays in receiving services and support (National Board of Health and Welfare, 2024). The shortage of human resources creates time pressure implying that service providers cannot spend enough time on each client. Women felt rushed during their interactions, leaving many feeling undervalued and unsupported, deterring future contact with IPV services.

Additionally, women reported experiencing power imbalance during their interactions with service providers. There was a sentiment among some participants that their input was disregarded, and they were not afforded the chance to articulate their problems or participate in determining the course of action. Service providers unwittingly perpetuate ableist biases and hierarchies of power in the provision of IPV services (Kudlick, 2005). Women further described a lack of empathy; some providers made no effort to hear and understand what the women communicated. This bias is deeply embedded in social practices where women with disabilities are confronted with marginalization, rejection, and extensive control over all aspects of their lives (Chenoweth, 1996).

Women were dissuaded from seeking IPV services due to professional misconduct which manifested in breach of confidentiality and failure to adhere to the standard response. Some women were discouraged from seeking professional help because of the absence of justice and retribution for perpetrators. It should be noted that the lack of justice further legitimizes violence (Overstreet & Quinn, 2013). Previous research calls for institutions to establish training programs and service protocols to guide responses to victims of IPV with disabilities (Garcia-Moreno, 2002). In recent years, the Swedish government through the National Board of Health and Welfare, issued guidelines to aid institutional response to violence (SOU, 2014). However, these are general guidelines, with no specific attention to the needs of women with disabilities. IPV service providers noted that having targeted guidelines was instrumental in offering support to women with disabilities (Namatovu & Ineland, 2024).

### Methodological Considerations

In the methods section, we specify that a reflective thematic analysis was performed, we admit to our subjectivity as researchers owning our role as active agents in the production of knowledge. Braun and Clarke (2012) view subjectivity as a fundamental resource that allows researchers to create contextualized meanings and knowledge. The awareness of own biases and perspectives led to more nuanced interpretations of data. However, it is possible that our personal beliefs and experiences might have influenced our interpretation.

When conducting in-depth interviews, an interview guide with a pre-determined set of questions was used to enable us to obtain detailed accounts of the participants. We framed and presented these questions as open-ended, allowing participants to explore our pre-set topics and room to provide additional dimensions they deemed relevant. This approach enables exploration of the participants’ subjective experiences and sense-making (Braun & Clarke, 2021). When using reflective thematic analysis subjectivity is an asset, as it aligns with the researchers’ goal of drawing attention to the subjective meanings and experiences of the participants (Braun & Clarke, 2023).

Another aspect worthy of discussion is the sample size, which comprised of 11 women who participated in the interviews. We consider this number sufficient to address our need to identify qualitatively meaningful patterns rather than to quantify magnitudes (Braun & Clarke, 2012). We reached out directly to the population of people with disabilities through advertising on social media and magazines directed at this population. In addition, we requested IPV service providers who met women with disabilities to inform them of our research and to provide the project contact details for those who showed interest. Recruitment lasted a period of 10 months, data was collected during the COVID pandemic when all physical meeting places we closed, making it even harder to reach the target population.

Lastly, it should be noted that we only interviewed participants who could independently give verbal and written informed consent. Therefore, our sample could be viewed as a selective sample, even within the population of women with disabilities. However, we had to abide by this inclusion restriction because it was a requirement from the ethical review board. Ethics committees put in place such strict guidelines to protect individuals perceived as vulnerable (Carlson, 2013). However, this could be an overestimation of risks which could lead to further exclusion of women with intellectual disabilities, reinforcing underrepresentation of this population in research.

## Conclusion

This study indicates that women with disabilities are confronted with several challenges that hinder access to IPV services. Achieving optimal access requires addressing stigma attached to IPV and disability, examining the role of social networks and addressing institutional gaps that prevent women with disabilities from seeking IPV services. There is a need for institutions to address stigma and offer appropriate training to service providers. Additionally, increased resource investment in IPV systems could help address insufficient human resource and thereby combat delays and long waiting time. This study contributes to an area of research that has received minimal attention globally and in Sweden. By adding the authentic voices of women with disabilities, the study provides a perspective beyond the dominant bio-medical cultural paradigm. Failure to include this population re-enforces a research culture of using proxy voices thereby denying women with disabilities the power to be heard as expert witnesses of their own experiences. This study provides a robust set of findings while calling upon researchers across disciplines to generate more knowledge geared at improving access to IPV services for women with disabilities.

## Supplemental Material

sj-pdf-1-vaw-10.1177_10778012251379425 - Supplemental material for “Struggles and Falling Between the Cracks”: Experiences of Women With Disabilities on Using Intimate Partner Violence Services Supplemental material, sj-pdf-1-vaw-10.1177_10778012251379425 for “Struggles and Falling Between the Cracks”: Experiences of Women With Disabilities on Using Intimate Partner Violence Services by Fredinah Namatovu, Veronica Lövgren and Kim Wickman in Violence Against Women

## References

[bibr1-10778012251379425] Åker T. H. Moen K. Josefsson K. A. Frawley P. (2024). Empowering healthcare professionals: Exploring experiences leading a violence prevention course for adults with intellectual disability. Intellectual and Developmental Disabilities, 62(5), 363–375. 10.1352/1934-9556-62.5.363. PMID: 39317371 39317371

[bibr2-10778012251379425] Anyango C. Goicolea I. Namatovu F. (2023). Women with disabilities’ experiences of intimate partner violence: A qualitative study from Sweden. BMC Women's Health, 23(1), Article 381. 10.1186/s12905-023-02524-8 PMC10360297 37474929

[bibr3-10778012251379425] Berns N. (2001). Degendering the problem and gendering the blame: Political discourse on women and violence. Gender & Society, 15(2), 262–281. 10.1177/089124301015002006

[bibr4-10778012251379425] Bogart K. R. Dunn D. S. (2019). Ableism special issue Introduction. Journal of Social Issues, 75(3), 650–664. 10.1111/josi.12354

[bibr5-10778012251379425] Bowes A. M. Domokos T. M. (1993). South Asian women and health services: A study in Glasgow. New Community, 19(4), 611–626. 10.1080/1369183X.1993.9976391

[bibr6-10778012251379425] Braun V. Clarke V. (2006). Using thematic analysis in psychology. Qualitative Research in Psychology, 3(2), 77–101. 10.1191/1478088706qp063oa

[bibr7-10778012251379425] Braun V. Clarke V. (2012). Thematic analysis. In APA Handbook of research methods in psychology, vol 2: Research designs: Quantitative, qualitative, neuropsychological, and biological (pp. 57–71). American Psychological Association. 10.1037/13620-004

[bibr8-10778012251379425] Braun V. Clarke V. (2019). Reflecting on reflexive thematic analysis. Qualitative Research in Sport, Exercise and Health, 11(4), 589–597. 10.1080/2159676X.2019.1628806

[bibr9-10778012251379425] Braun V. Clarke V. (2021). One size fits all? What counts as quality practice in (reflexive) thematic analysis? Qualitative Research in Psychology, 18(3), 328–352. 10.1080/14780887.2020.1769238

[bibr10-10778012251379425] Braun V. Clarke V. (2022). Thematic analysis: A practical guide. Sage publications.

[bibr11-10778012251379425] Braun V. Clarke V. (2023). Toward good practice in thematic analysis: Avoiding common problems and be(com)ing a knowing researcher. International Journal of Transgender Health, 24(1), 1–6. 10.1080/26895269.2022.2129597 36713144 PMC9879167

[bibr12-10778012251379425] Brownridge D. A. (2006). Partner violence against women with disabilities: Prevalence, risk, and explanations. Violence Against Women, 12(9), 805–822. 10.1177/1077801206292681 16905674

[bibr13-10778012251379425] Campbell F. A. K. (2008). Exploring internalized ableism using critical race theory. Disability & Society, 23(2), 151–162. 10.1080/09687590701841190

[bibr14-10778012251379425] Canfield A. (2007). Stephen king's dolores claiborne and rose madder: A literary backlash against domestic violence. Journal of American Culture (Malden, Mass.), 30(4), 391–400. 10.1111/j.1542-734X.2007.00616.x

[bibr15-10778012251379425] Carlson L. (2013). Research ethics and intellectual disability: Broadening the debates. Yale Journal of Biology and Medicine, 86(3), 303–314. 24058305 PMC3767215

[bibr16-10778012251379425] Chenoweth L. (1996). Violence and women with disabilities: Silence and paradox. Violence Against Women, 2(4), 391–411. 10.1177/1077801296002004004

[bibr17-10778012251379425] Coleman J. S. (1990). Foundations of social theory. Harvard University Press.

[bibr18-10778012251379425] Coleman L. (1997). Stigma: An enigma demystified. Routledge.

[bibr19-10778012251379425] Crenshaw K. (1991). Mapping the margins: Intersectionality, identity politics, and violence against women of color. Stanford Law Review, 43(6), 1241–1299. 10.2307/1229039

[bibr20-10778012251379425] Cureton A. (2018). Hiding a disability and passing as non-disabled. In Cureton A. Hill J. T. E. (Eds.), Disability in practice: Attitudes, policies and relationships (pp. 18–32). Oxford University Press.

[bibr21-10778012251379425] Dufort M. Gumpert C. H. Stenbacka M. (2013). Intimate partner violence and help-seeking a cross-sectional study of women in Sweden. BMC Public Health, 13, 866. 10.1186/1471-2458-13-866 24053735 PMC3852487

[bibr22-10778012251379425] Ellsberg M. Jansen H. A. F. M. Heise L. Watts C. H. Garcia-Moreno C. (2008). Intimate partner violence and women's physical and mental health in the WHO multi-country study on women's health and domestic violence: An observational study. The Lancet, 371(9619), 1165–1172. 10.1016/S0140-6736(08)60522-X 18395577

[bibr23-10778012251379425] Evans M. A. Feder G. S. (2016). Help-seeking amongst women survivors of domestic violence: A qualitative study of pathways towards formal and informal support. Health Expectations: An International Journal of Public Participation in Health Care and Health Policy, 19(1), 62–73. 10.1111/hex.12330 25556776 PMC5055220

[bibr24-10778012251379425] Feder G. S. Hutson M. Ramsay J. Taket A. R. (2006). Women exposed to intimate partner violence: Expectations and experiences when they encounter health care professionals: A meta-analysis of qualitative studies. Archives of Internal Medicine, 166(1), 22–37. 10.1001/archinte.166.1.22 16401807

[bibr25-10778012251379425] Fleming J. C. Franklin C. A. (2021). Predicting police endorsement of myths surrounding intimate partner violence. Journal of Family Violence, 36, 407–416. 10.1007/s10896-020-00178-w

[bibr26-10778012251379425] Follingstad D. R. DeHart D. D. (2000). Defining psychological abuse of husbands toward wives: Contexts, behaviors, and typologies. Journal of Interpersonal Violence, 15(9), 891–920. 10.1177/088626000015009001

[bibr27-10778012251379425] Garcia-Moreno C. (2002). Dilemmas and opportunities for an appropriate health-service response to violence against women. Lancet, 359(9316), 1509–1514. 10.1016/S0140-6736(02)08417-9 11988263

[bibr28-10778012251379425] Garcia-Moreno C. Jansen H. A. Ellsberg M. Heise L. Watts C. H. , & Health, WHO Multi-country Study on Women’s Health and Domestic Violence against Women Study . (2006). Prevalence of intimate partner violence: Findings from the WHO multi-country study on women's health and domestic violence. Lancet, 368(9543), 1260–1269. 10.1016/S0140-6736(06)69523-8 17027732

[bibr29-10778012251379425] Goffman E. (1959). The presentation of self in everyday life. Doubleday.

[bibr30-10778012251379425] Goffman E. (1963). Stigma: Notes on the Management of Spoiled Identity. Englewood Cliffs.

[bibr31-10778012251379425] Guba E. G. Lincoln Y. S. (1994). Competing paradigms in qualitative research. In Handbook of qualitative research (pp. 105–117). Sage Publications, Inc.

[bibr32-10778012251379425] Heise L. L. (1998). Violence against women: An integrated, ecological framework. Violence Against Women, 4(3), 262–290. 10.1177/1077801298004003002 12296014

[bibr33-10778012251379425] Hughes K. Bellis M. A. Jones L. Wood S. Bates G. Eckley L. McCoy E. Mikton C. Shakespeare T. Officer A. (2012). Prevalence and risk of violence against adults with disabilities: A systematic review and meta-analysis of observational studies. Lancet, 379(9826), 1621–1629. 10.1016/S0140-6736(11)61851-5 22377290

[bibr34-10778012251379425] Jóhannsdóttir Á Egilson S. T. Gibson B. E. (2021). What’s shame got to do with it? The importance of affect in critical disability studies. Disability & Society, 36(3), 342–357. 10.1080/09687599.2020.1751076

[bibr35-10778012251379425] Jones L. Bellis M. A. Wood S. Hughes K. McCoy E. Eckley L. Bates G. Mikton C. Shakespeare T. Officer A. (2012). Prevalence and risk of violence against children with disabilities: A systematic review and meta-analysis of observational studies. Lancet, 380(9845), 899–907. 10.1016/S0140-6736(12)606928 22795511

[bibr36-10778012251379425] Klint F. Källström Å Farias L. (2023). Social work practices with victims of violence among people with cognitive disabilities. Nordic Social Work Research, 14(3), 317–331. 10.1080/2156857X.2023.2285980

[bibr37-10778012251379425] Kudlick C. J. (2005). Disability history, power, and rethinking the idea of “the other”. PMLA/Publications of the Modern Language Association of America, 120(2), 557–561. 10.1632/S0030812900167896

[bibr38-10778012251379425] Lawoko S. Sanz S. Helström L. Castren M. (2011). Screening for intimate partner violence against women in healthcare Sweden: Prevalence and determinants. ISRN Nursing, 2011, 510692. 10.5402/2011/510692 22254143 PMC3255304

[bibr39-10778012251379425] Leary K. (1999). Passing, posing, and “keeping it real”. Constellations (Oxford, England), 6(1), 85–96. 10.1111/1467-8675.00122

[bibr40-10778012251379425] Levesque J.-F. Harris M. F. Russell G. (2013). Patient-centred access to health care: Conceptualising access at the interface of health systems and populations. International Journal for Equity in Health, 12(1), 18. 10.1186/1475-9276-12-18 23496984 PMC3610159

[bibr41-10778012251379425] Lövestad S. Krantz G. (2012). Men’s and women’s exposure and perpetration of partner violence: An epidemiological study from Sweden. BMC Public Health, 12(1), 945. https://doi.org/Artn94510.1186/1471-2458-12-949 23116238 10.1186/1471-2458-12-945 PMC3534228

[bibr42-10778012251379425] Lövestad S. Vaez M. Löve J. Hensing G. Krantz G. (2021). Intimate partner violence, associations with perceived need for help and health care utilization: A population-based sample of women in Sweden. Scandinavian Journal of Public Health, 49(3), 268–276. 10.1177/1403494820930952 32854572 PMC8056709

[bibr43-10778012251379425] Lund E. M. (2011). Community-based services and interventions for adults with disabilities who have experienced interpersonal violence: A review of the literature. Trauma, Violence & Abuse, 12(4), 171–182. 10.1177/1524838011416377 21908437

[bibr44-10778012251379425] McRuer R. (2008). Crip theory. Cultural signs of queerness and disability. Scandinavian Journal of Disability Research, 10(1), 67–69. httpsss://doi.org/ http://doi.org/10.1080/15017410701880122 10.1080/15017410701880122

[bibr45-10778012251379425] Meyer S. (2012). Why women stay: A theoretical examination of rational choice and moral reasoning in the context of intimate partner violence. Australian & New Zealand Journal of Criminology, 45(2), 179–193. 10.1177/0004865812443677

[bibr46-10778012251379425] Meyer S. (2016). Still blaming the victim of intimate partner violence? Women’s narratives of victim desistance and redemption when seeking support. Theoretical Criminology, 20(1), 75–90. 10.1177/1362480615585399

[bibr47-10778012251379425] Meyer S. R. Mosha N. R. Shakespeare T. Kuper H. Mtolela G. Harvey S. Kapiga S. Mshana G. Stockl H. (2023). Disability and intimate partner violence: A cross-sectional study from mwanza, Tanzania. Disability and Health Journal, 16(2), 101404. 10.1016/j.dhjo.2022.101404 36522283

[bibr48-10778012251379425] Morrow S. L. (2005). Quality and trustworthiness in qualitative research in counseling psychology. Journal of Counseling Psychology, 52(2), 250–260. 10.1037/0022-0167.52.2.250

[bibr49-10778012251379425] Mueller C. O. Forber-Pratt A. J. Sriken J. (2019). Disability: Missing from the conversation of violence. Journal of Social Issues, 75(3), 707–725. 10.1111/josi.12339

[bibr50-10778012251379425] Murray C. E. Crowe A. Brinkley J. (2015). The stigma surrounding intimate partner violence: A cluster analysis study. Partner Abuse, 6(3), 320–336. 10.1891/1946-6560.6.3.320

[bibr51-10778012251379425] Namatovu F. Ineland J. (2024). Collaboration in providing intimate-partner violence services to women with disabilities. BMC Public Health, 24, 1863. 10.1186/s12889-024-19352-6 38992636 PMC11241963

[bibr52-10778012251379425] Namatovu F. Ineland J. Lövgren V. (2024). Exploring the perspectives of professionals on providing intimate partner violence services to women with disabilities. Violence Against Women, 30(2), 622–640. 10.1177/10778012221137916 36408719 PMC10775642

[bibr53-10778012251379425] Namatovu F. Preet R. Goicolea I. (2018). Gender-based violence among people with disabilities is a neglected public health topic. Global Health Action, 11, 97–100. 10.1080/16549716.2019.1694758 PMC8820249 31777318

[bibr54-10778012251379425] National Board of Health and Welfare . (2024). Proposal for a national plan for national healthcare agency: Interim report (in Swedish). ( https://www.socialstyrelsen.se/globalassets/sharepoint-dokument/artikelkatalog/ovrigt/2024-3-8999.pdf )

[bibr55-10778012251379425] Nosek M. A. Foley C. C. Hughes R. B. Howland C. A. (2001). Vulnerabilities for abuse among women with disabilities. Sexuality and Disability, 19(3), 177–189. 10.1023/a:1013152530758

[bibr56-10778012251379425] Overstreet N. M. Quinn D. M. (2013). The intimate partner violence stigmatization model and barriers to help seeking. Basic and Applied Social Psychology, 35(1), 109–122. 10.1080/01973533.2012.746599 23524454 PMC3601798

[bibr57-10778012251379425] Pugh B. Li L. Sun I. Y. (2021). Perceptions of why women stay in physically abusive relationships: A comparative study of Chinese and U.S. College students. Journal of Interpersonal Violence, 36(7–8), 3778–3813. 10.1177/0886260518778264 29808779

[bibr58-10778012251379425] Robinson S. Frawley P. Dyson S. (2021). Access and accessibility in domestic and family violence services for women with disabilities: Widening the Lens. Violence Against Women, 27(6–7), 918–936. https://doi.org/Artn 1077801220909890 32339075 10.1177/1077801220909890

[bibr59-10778012251379425] Rose L. E. Campbell J. Kub J. (2000). The role of social support and family relationships in women's responses to battering. Health Care for Women International, 21(1), 27–39. 10.1080/073993300245384 11022447

[bibr60-10778012251379425] Seff L. R. Beaulaurier R. L. Newman F. L. (2008). Nonphysical abuse: Findings in domestic violence against older women study. Journal of Emotional Abuse, 8(3), 355–374. 10.1080/10926790802278933

[bibr61-10778012251379425] Shah S. Tsitsou L. Woodin S. (2016). Hidden voices: Disabled women’s experiences of violence and support over the life course. Violence Against Women, 22(10), 1189–1210. 10.1177/1077801215622577 26762144

[bibr62-10778012251379425] Skoog Waller S. (2022). *Utan mig är du helt ensam: kvinnors levda erfarenheter av omgivningens och samhällets ensamgörande i spåren av mäns våld och eftervåld*. http://urn.kb.se/resolve?urn=urn:nbn:se:hig:diva-38020

[bibr63-10778012251379425] SOU - Staten Offentliga Utledningar . (2014). Violence in close relationships: A public health issue. https://www.regeringen.se/rattsliga-dokument/statens-offentliga-utredningar/2014/06/sou-201449/

[bibr64-10778012251379425] Stark E. (2007). Coercive control: How men entrap women in personal life. Oxford University Press.

[bibr65-10778012251379425] Starke M. Larsson A. Punzi E. (2024). People with intellectual disability and their risk of exposure to violence: Identification and prevention – a literature review. Journal of Intellectual Disabilities, 29(3), 760–783. 10.1177/17446295241252472 38714505 PMC12397523

[bibr66-10778012251379425] Thomas C. (1999). Female forms: experiencing and understanding disability. Open University Press.

[bibr67-10778012251379425] Thomas K. A. Joshi M. Wittenberg E. McCloskey L. A. (2008). Intersections of harm and health: A qualitative study of intimate partner violence in women's lives. Violence Against Women, 14(11), 1252–1273. 10.1177/1077801208324529 18809846

[bibr68-10778012251379425] Valentine A. Akobirshoev I. Mitra M. (2019). Intimate partner violence among women with disabilities in Uganda. International Journal of Environmental Research & Public Health, 16(6), 947. 10.3390/ijerph16060947 30884787 PMC6466247

[bibr69-10778012251379425] World Health Organisation . (2013). Responding to intimate partner violence and sexual violence against women: WHO clinical and policy guidelines. World Press. Responding to intimate partner violence and sexual violence against women (who.int) 24354041

